# Cytosolic GAPDH as a redox-dependent regulator of energy metabolism

**DOI:** 10.1186/s12870-018-1390-6

**Published:** 2018-09-06

**Authors:** Markus Schneider, Johannes Knuesting, Oliver Birkholz, Jürgen J. Heinisch, Renate Scheibe

**Affiliations:** 1 Division of Plant Physiology, Department of Biology and Chemistry, Osnabrück University, Barbarastr. 11, 49076 Osnabrück, Germany; 2 Division of Biophysics, Department of Biology and Chemistry, Osnabrück University, Barbarastr. 11, 49076 Osnabrück, Germany; 3 Division of Genetics, Department of Biology and Chemistry, Osnabrück University, Barbarastr. 11, 49076 Osnabrück, Germany

**Keywords:** Redox, Metabolism, GAPDH, VDAC, Thioredoxin, Mitochondria, Nucleus, Glycolysis, Metabolon, GapN

## Abstract

**Background:**

Plant cytosolic NAD-dependent glyceraldehyde-3-phosphate dehydrogenase (GapC) displays redox-dependent changes in its subcellular localizations and activity. Apart from its fundamental role in glycolysis, it also exhibits moonlighting properties. Since the exceptional redox-sensitivity of GapC has been suggested to play a crucial role in its various functions, we here studied its redox-dependent subcellular localization and the influence of the redox-state on GapC protein interactions.

**Results:**

In mesophyll protoplasts from *Arabidopsis thaliana,* colocalization of GapC with mitochondria was more pronounced under reducing conditions than upon oxidative stress. In accordance, reduced GapC showed an increased affinity to the mitochondrial voltage-dependent anion-selective channel (VDAC) compared to the oxidized one*.* On the other hand, nuclear localization of GapC was increased under oxidizing conditions. The essential role of the catalytic cysteine for nuclear translocation was shown by using the corresponding cysteine mutants. Furthermore, interaction of GapC with the thioredoxin Trx-*h3* as a candidate to revert the redox-modifications, occurred in the nucleus of oxidized protoplasts. In a yeast complementation assay, we could demonstrate that the plant-specific non-phosphorylating glyceraldehyde 3-P dehydrogenase (GapN) can substitute for glucose 6-P dehydrogenase to generate NADPH for re-reduction of the Trx system and ROS defense.

**Conclusions:**

The preferred association of reduced, glycolytically active GapC with VDAC suggests a substrate-channeling metabolon at the mitochondrial surface for efficient energy generation. Increased occurrence of oxidized GapC in the nucleus points to a function in signal transduction and gene expression. Furthermore, the interaction of GapC with Trx-*h3* in the nucleus indicates reversal of the oxidative cysteine modification after re-establishment of cellular homeostasis. Both, energy metabolism and signal transfer for long-term adjustment and protection from redox-imbalances are mediated by the various functions of GapC. The molecular properties of GapC as a redox-switch are key to its multiple roles in orchestrating energy metabolism.

**Electronic supplementary material:**

The online version of this article (10.1186/s12870-018-1390-6) contains supplementary material, which is available to authorized users.

## Background

Pathways of plant metabolism that generate energy equivalents both from light and from substrate in the various cellular compartments must be permanently adjusted to changing environmental factors and metabolic requirements. The electron-transport chains in chloroplasts and mitochondria are major sources of redox-imbalances and radical formation under stress. Signals arising from such imbalances are sensed and transferred to the nucleus. Altered gene expression will then help to maintain homeostasis of metabolic fluxes and cellular redox-state [1].

NAD-dependent glyceraldehyde-3-phosphate dehydrogenase (GAPDH; E.C. 1.2.1.12) is a highly conserved glycolytic enzyme in all kingdoms of life. Several non-glycolytic functions of GAPDH have also been observed, classifying this enzyme as prototypical example for moonlighting (for reviews see [2–6]:). In *A. thaliana*, GapC1 (At3g04120) and GapC2 (At1g13440) are the GAPDH isoforms residing in the cytosol. We previously characterized redox-dependent cysteine modifications of GapC, namely *S*-glutathionylation and *S*-nitrosylation, which result in inactivation of the enzymes [7]. In a yeast two-hybrid screen, VDAC was identified as an interaction partner of GapC [8]. Moreover, a fraction of GapC was shown to be localized to the nucleus of Arabidopsis mesophyll protoplasts [7, 9–11], and associated with the outer mitochondrial membrane (OMM) and the cytoskeleton [9]. S-sulfhydrylation of GapC has been observed to also result in nuclear localization of the modified protein [12].

GAPDH is most sensitive towards oxidation at its catalytic cysteine residue [13–15], and thus represents an initial redox sensor and a hub to induce the various responses that are required to maintain redox-homeostasis [15, 16]. Glutathionylation and nitrosylation of GapC were found in large-scale proteomic approaches, both in plants and animals [17–20]. GAPDH was also suggested to act as an H_2_O_2_ sensor by triggering the protective oxidative stress response and re-establishment of cellular homeostasis [21]. Modification of the catalytic cysteine of GapC, only occurring in the absence of the substrate, leads to reversible or irreversible inactivation of the enzyme, depending on the oxidant, as demonstrated in vitro [7]. In addition to the regulation of enzyme activities, such modifications are assumed to affect signaling cascades and other moonlighting functions.

Trx and NADPH-thioredoxin reductases (NTR) are universal mediators of cellular redox-modifications [22, 23]. For continuous re-reduction, reducing equivalents can be provided by NADPH-dependent reductases, e.g., glucose-6-P dehydrogenase (G6PDH) or the plant-specific irreversible non-phosphorylating glyceraldehyde-3 phosphate reductase (GapN; EC 1.2.1.9) [24].

In this study, we focused on the redox-dependent interactions of GapC with VDAC as a central regulator of the metabolic flux across the OMM [25, 26], as well as the nuclear translocation of GapC, and its interaction with Trx-*h3* as a candidate for catalyzing the reversal of the transient oxidative modification [27]. We used both, in vitro approaches such as reflectance interferometry (RIf) as well as in vivo experiments by expressing fluorescent fusion proteins in isolated mesophyll protoplasts and by heterologous enzyme complementation in yeast. Oxidants and reductants, as well as cysteine mutants of GapC, were used to analyze the redox-dependent localization of GapC1 and GapC2 in the different cellular subcompartments and their interactions with VDAC3 and Trx-*h3*. The cellular localizations and interactions found under reducing and under oxidizing conditions might help to unravel the various functions of GapC in more detail.

## Methods

### Isolation and transient transformation of Arabidopsis protoplasts

Subcellular localization and interaction studies with fluorescent fusion proteins of GapC1, GapC2 and Trx-*h3* were performed in mesophyll protoplasts isolated from leaves of 4-week-old wild-type *A. thaliana* plants grown under short-day conditions. Isolation and transformation of protoplasts were performed according to [28], with modifications described in [29].

### Imaging of fluorescent fusion proteins in transiently transformed protoplasts

For GapC-localization studies, SmaI/XbaI fragments encoding C-terminal mEGFP-fusions of GapC1 and GapC2 were cloned into the binary transformation vector pBAR-35S. To express the fusion proteins in vivo, mesophyll protoplasts were transiently transformed with the pBAR-35S-mEGFP constructs and incubated for 16 h at 22 °C. Plant mitochondria were visualized by staining with 500 nM MitoTracker® Red FM (Thermo Fisher Scientific, Waltham, USA). The protoplasts were imaged using a confocal laser scanning microscope (cLSM) 510META (Carl Zeiss, Göttingen, Germany). The mEGFP fluorescence and chlorophyll autofluorescence were excited at 488 nm. The mEGFP emission was detected at 500–530 nm, chlorophyll autofluorescence was monitored in the range of 650–700 nm.

To quantify the nuclear localization of GapC1 and GapC2 fluorescent fusions, the basic leucine zipper protein bZIP63 was used as a nuclear marker protein. The bZIP63 cDNA from Arabidopsis was cloned as a SmaI/XbaI fragment into the binary vector pBAR-35S to yield a C-terminal mCherry fusion. The resulting plasmid was co-transformed with constructs encoding GapC-mEGFP fusion proteins, as indicated. The mCherry fluorescence was detected by excitation at 585 nm and emission at 600–620 nm. The mEGFP signal was determined in the cytosol and the nucleus for at least 50 protoplasts in three independent experiments as described above. The fluorescence intensities of the cytosolic and nuclear mEGFP signals were calculated as the ratio of each individual signal to the sum of both.

For split-YFP experiments, the cDNA sequences of the Arabidopsis proteins GapC1, GapC2 and Trx-*h3* were cloned as BamHI/KpnI fragments into the vectors pUC-SPYNE or pUC-SPYCE [30] to generate C-terminal fusions with the N- or C-terminal part of YFP, respectively. bZIP63-mCherry was used as a nuclear marker as described above. To express the fusion proteins in vivo, mesophyll protoplasts were transiently co-transformed with the pBAR-35S constructs and incubated for 16 h at 22 °C. For detection, complemented YFP was excited at 514 nm, and emission was monitored at 525–540 nm.

For each experiment, cLSM raw data were processed with the Fiji software system [31].

### Treatments of isolated protoplasts with redox-reagents

To analyze localization and interactions of the various proteins under different redox conditions, transformed Arabidopsis mesophyll protoplasts were treated with 10 mM DTT, 50 μM GSNO or 0.2 mM H_2_O_2_ by incubation at 22 °C for 40 min prior to cLSM analysis. In preliminary experiments, intactness of the protoplasts after the treatments was checked.

### Expression and purification of recombinant GapC and Trx-*h3* from *A. thaliana*

Expression and purification of the recombinant GapC and Trx-*h3* proteins from *A. thaliana* were performed as previously described [7]. In the case of Trx-*h3,* the protein was stored in 20 mM Tris-HCl (pH 8.0) containing 150 mM NaCl. GapC was stored in 50 mM Tris-HCl (pH 7.8), 5 mM EDTA, 150 mM KCl, 2 mM DTT, 140 μM NAD, 50% glycerol, and desalted before use.

### Expression, refolding, purification and reconstitution of recombinant VDAC3 from *A. thaliana*

Overexpression, refolding and purification of Arabidopsis VDAC3 was performed according to [32, 33] with modifications. VDAC3 was overexpressed using the cold-shock expression system described by [34]. The Arabidopsis VDAC3 cDNA was cloned into the pCold I DNA vector (Takara Bio Europe/Clontech, Saint-Germain-en-Laye, France) using SacI and XbaI. The *E. coli* mutant strain BL21(DE3)-omp9 [F^−^*, ompT hsdS*_*B*_ *(r*_*B*_^*−*^ *m*_*B*_^*−*^*) gal dcm (DE3) ΔlamB ompF::Tn5 ΔompA ΔompC ΔompN::*Ω] [35, 36] was transformed with the pCold I DNA-AtVDAC3 vector. Liquid LB medium (containing 100 μg ml^− 1^ ampicillin) was inoculated with a starter culture. Cells were cultivated at 37 °C under shaking at 200 rpm. After reaching an OD_600_ of 0.8–1.0 the cultures were incubated on ice for 30 min. Protein expression was induced by the addition of isopropyl-β-D-thiogalactopyranoside (1 mM) and transfer of the cultures to 15 °C. The cells were incubated at 15 °C and 200 rpm for further 24 h and finally harvested by centrifugation (5000 x*g*, 20 min, 4 °C). The cell pellet was shock-frozen in liquid nitrogen and stored at − 80 °C. For protein purification, the cells were thawed, emerged in resuspension buffer (50 mM Tris-HCl, pH 8.0, 100 mM NaCl, 0.1 mM EDTA) and disrupted by ultrasonication (Vibracell, Fisher Scientific, Illkirch, France). The VDAC3-containing inclusion bodies were collected by centrifugation (25000 x*g*, 30 min, 4 °C), and washed three times with detergent-containing washing buffer (50 mM Tris-HCl, pH 8.0, 100 mM NaCl, 10 mM EDTA, 2.5% (*v*/v) Triton X-100), followed by three washing steps using detergent-free washing buffer (50 mM Tris-HCl, pH 8.0, 100 mM NaCl). The inclusion bodies were treated with denaturation buffer (25 mM NaH_2_PO_4_/Na_2_HPO_4_, pH 7.0, 100 mM NaCl, 1 mM EDTA, 10 mM DTT, 6 M guanidinium chloride). A 10-fold dilution of the guanidinium chloride was achieved by drop-wise addition to refolding buffer I (25 mM NaH_2_PO_4_/Na_2_HPO_4_, pH 7.0, 100 mM NaCl, 1 mM EDTA, 2.22% (*v*/v) lauryl-dimethyl-amine oxide (LDAO)). This mixture was stirred for 18 h at 4 °C. A further 10-fold dilution of the guanidinium chloride by drop-wise addition to refolding buffer II (25 mM NaH_2_PO_4_/Na_2_HPO_4_, pH 7.0, 10 mM NaCl, 1 mM EDTA, 0.1% (v/v) LDAO) and stirring for 18 h at 4 °C followed this step.. Insoluble material was removed by passage through a 0.22-μm filter. For cation-exchange chromatography, the sample was loaded onto a pre-equilibrated Fractogel® EMD SE Hicap (M) (Merck, Darmstadt, Germany) cation-exchange column connected to an ÄKTAprime plus chromatography system (GE Healthcare, Munich, Germany). Elution of the refolded VDAC3 proteins was achieved using a linear gradient from 10 mM NaCl to 1 M NaCl in the elution buffer (25 mM NaH_2_PO_4_/Na_2_HPO_4_, pH 8.0, 10 mM NaCl, 1 mM EDTA, 1 mM DTT, 0.1% (*v*/v) LDAO). The VDAC3-containing fractions were pooled and concentrated using a 10-kDa Amicon concentrator (Merck, Darmstadt, Germany). Size-exclusion chromatography was performed by loading the refolded VDAC3 onto pre-equilibrated (10 mM Tris-HCl, pH 8.0, 100 mM NaCl, 0.05% LDAO) Superose™ 12 10/300 GL column (GE Healthcare, Munich, Germany), and the elution profiles were recorded at 280 nm. The protein-containing fractions concentrated using a 10-kDa Amicon concentrator (Merck, Darmstadt, Germany).

### Preparation of VDAC3-containing proteoliposomes

For RIf experiments, the recombinant VDAC3 was reconstituted into 1,2-dioleoyl-*sn*-glycero-3-phosphocholine (DOPC) liposomes in a molar ratio of 1:1000 (protein:lipid). The VDAC3- containing proteoliposomes were formed by detergent extraction in the presence of heptakis-(2,6-di-O-methyl)-*β*-cyclodextrin (*β*-CD) (Sigma) [37]. A mixture of 5 mM of DOPC and 20 mM Triton X-100 in HBS buffer (20 mM Hepes-KOH, pH 7.4, 300 mM NaCl) and LDAO-solubilized recombinant VDAC3 was prepared before a 2-fold excess of *β*-CD over the detergent was added. The mixture was incubated for 5 min at room temperature and diluted in HBS buffer.

### Reflectance interferometry (RIf)

Poly-L-lysine (PLL − PEG-AC) modification of glass coverslips required for RIf was performed as described by [38, 39]. All measurements were conducted in a home-built setup as described in detail by [40]. The polymer-supported membrane was formed by injection of the VDAC3-containing proteoliposomes followed by rinsing with HBS buffer. After formation of the polymer-supported membrane, pre-treated recombinant GapC was injected. For each GapC injection, the surface was regenerated by two washing steps with 0.1% (*v*/v) Triton X-100. Extraction of pseudo-first order kinetic constants was performed by separately fitting dissociation and association phases using BIAevaluation 3.1 software (GE Healthcare, Little Chalfont, UK).

Silanization of RIf transducer chips with (3-glycidyl oxypropyl)-trimethoxysilane, and subsequent reaction with bis-amino-PEG with an average molecular mass of 2,000 Da was carried out as previously described [41]. Terminal amines were converted into carboxylates by incubation with 1 M succinic anhydride in 20 mM Hepes-KOH, pH 7.0, before the surface was NHS-activated by incubation with equimolar amounts of N-hydroxysuccinimide and 1-ethyl-3-(3-dimethylaminopropyl) carbodiimide (each 500 mM in dimethylformamide (DMF)) for 30 min. After washing with DMF, recombinant GapC1 at μmolar concentrations was added for immobilization. After 15 min at room temperature, excess protein was removed by rinsing with HBS buffer.

### Yeast strains and constructs for heterologous expression of the GapN encoding gene

To study phenotypic rescue in yeast, a derivative of the strain HD56-5A [42], was employed which carries a deletion of the sole *ZWF1* gene encoding glucose-6 phosphate dehydrogenase. To obtain this deletion, the one-step gene deletion method using a PCR product obtained from the template pUG6 [43] with the primer pair 16.235/16.236 (Additional file 1: Table S1) was applied to the homozygous diploid strain DHD5 [44]. Sporulation and tetrad analysis yielded the haploid segregant HOD269-1C (*MATa ura3–52 his3–11, 15 leu2–3,112 zwf1::kanMX*), which was used as a recipient strain for transformation with different plasmids as indicated in the results section. As a basis for heterologous gene expression, the vector pJJH2064 was used, which carries an optimized yeast *PFK2* promoter on a 576 bp EcoRI/BamHI DNA fragment obtained from custom-made string synthesis (Geneart, Thermo Fisher Scientific, Waltham, USA) introduced into the *CEN/ARS* plasmid YCplac33 [45]. The promoter sequence was chosen according to previous analyses indicating that it allows for full and constitutive expression of downstream genes [46]. Point mutations were inserted as required to eliminate internal restriction sites from the native promoter sequence. String synthesis was also employed to obtain the sequence of the Arabidopsis gene encoding GapN adapted to the yeast codon usage and flanked by a BamHI restriction site immediately preceding the ATG translation start codon and a HindIII site following the respective translation stop codons. The gene was then cloned as BamHI/HindIII-fragment into pJJH2064, which was introduced into the yeast recipient strain by selecting for uracil prototrophy. As controls, the vector not carrying an insertion, YCplac33 carrying the native yeast *ZWF1* gene including its flanking regions obtained by PCR with the primer pair 16.232/16.233 (Additional file 1: Table S1) from genomic yeast DNA cloned as a SacI/SalI fragment, or the yeast *ZWF1* gene introduced into pJJH2064 under the control of the *PFK2* promoter as a BamHI/HindIII fragment obtained with the oligonucleotide pair 16.234/16.233 (Additional file 1: Table S1) from genomic yeast DNA were used.

To assess the capacity of the different constructs to rescue the oxidative-stress sensitive phenotype of the yeast *zwf1* deletion strain, transformants were grown overnight in 3 ml of synthetic complete medium lacking uracil (SCura; 2% glucose, 0.67% yeast nitrogen base with ammonium sulfate, supplemented with standard amino acids and adenine) according to [47], diluted to an OD_600_ of approximately 0.1 in fresh medium and grown for another 4–6 h. Cells were again diluted to an OD_600_ of 0.1 and used to prepare four 10-fold serial dilutions. Five μl of each dilution was spotted on SCura plates containing 1.5% agar with H_2_O_2_ added at the concentrations as indicated. Growth was recorded by scanning the plates after 3 days of incubation at 30 °C.

For determination of specific enzyme activities, crude extracts were prepared from 20 ml of yeast cells growing exponentially in SCura at 30 °C. Cells were harvested, washed with 50 mM Tris-HCl, pH 7.5, and broken with glass beads as described previously [48]. Protein concentrations in crude extracts were determined with the Microbiuret method [49] using bovine serum albumin as a standard. G6PDH activity was measured by following NADP reduction at 340 nm after addition of 2.5 mM glucose 6-phosphate as the substrate to the reaction mixture, which consisted of 50 mM Tris-HCl, pH 7.5, and 0.4 mM NADP and appropriate amounts of crude extract. Similarly, GapN activity was determined in the same buffer by recording NADP reduction. To the latter, 0.5 U/ml aldolase was added as an ancillary enzyme, and the reaction was started with 2.5 mM fructose 1,6-bisphosphate.

## Results

### Association of GapC1 with mitochondria under different redox conditions

**Fig. 1:**
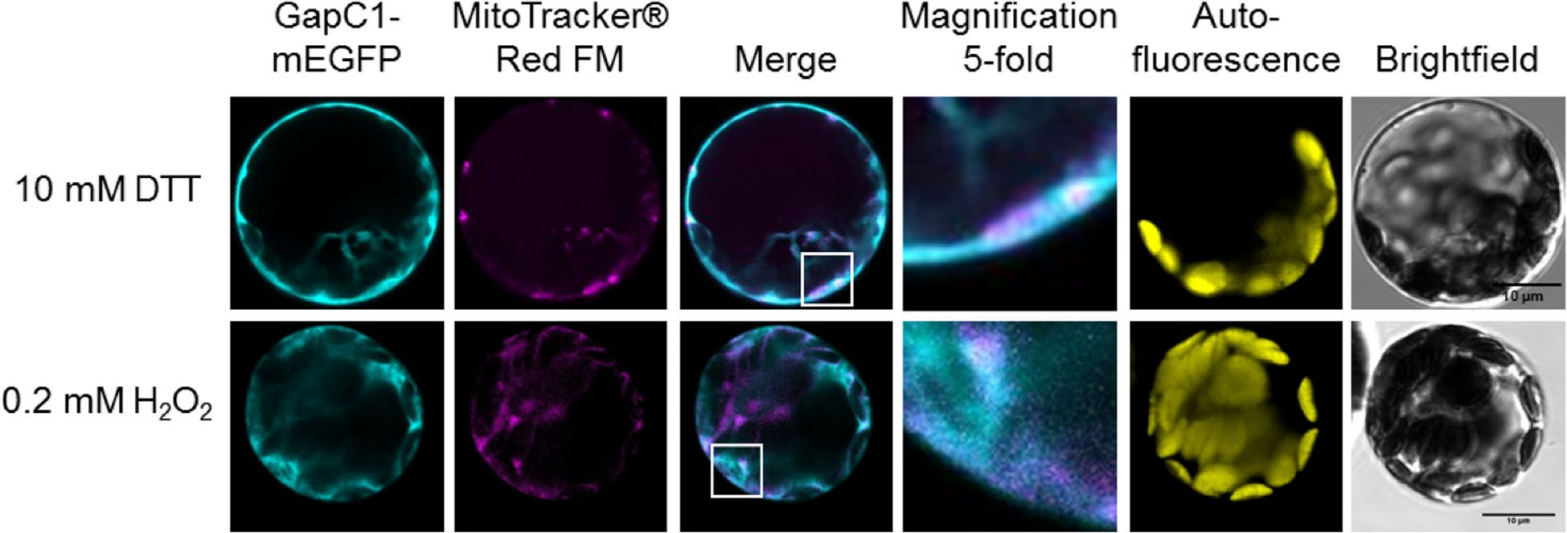
Effect of the redox-state on colocalization of GapC1 with mitochondria in Arabidopsis protoplasts. Protoplasts were transiently transformed with p35S-GapC1-mEGFP. Mitochondrial staining was performed by incubating the protoplasts in 500 nM MitoTracker® Red FM for 40 min. The protoplasts were pre-treated with 10 mM DTT or 0.2 mM H_2_O_2_ for 30 min to establish defined oxidizing and reducing conditions. Fluorescence signals are displayed in false colors: GapC1-mEGFP (cyan), MitoTracker® Red FM (magenta); chlorophyll autofluorescence (yellow). To estimate a tendency for the occurrence of colocalization, protoplasts (*n* ≥ 50) wereinspected. A representative image is shown for each condition.and Colocalization events are visualized in the merged picture as white regions. The scale bar is set to 10 μm

The GapC protein was previously found to associate with plant mitochondria [50, 51]. However, the role of this interaction remained unclear. Since metabolic fluxes and the cellular redox-state are closely related, we first analyzed the redox-dependence of the co-localization between GapC and mitochondria. For this purpose, isolated Arabidopsis mesophyll protoplasts were transformed with a GapC1-mEGFP fusion construct under the control of the S35-promoter. Simultaneously, mitochondria were stained with MitoTracker® Red FM to detect colocalizations. To assess the influence of the different redox conditions, protoplasts were pretreated with 0.2 mM H_2_O_2_ or 10 mM DTT. Fluorescent signals resulting from the fusion-proteins were detectable in the cytosol under reducing and oxidizing conditions (Fig. 1). Colocalization of GapC1-mEGFP with mitochondria was also found under both conditions, as indicated by white spots in the merged images. However, GapC1 was found to colocalize more frequently with mitochondria in a reducing environment, after DTT treatment of the protoplasts, than in the oxidized sample (H_2_O_2_ treatment) (Fig. 1). These findings suggest that the GapC1 protein is predominantly recruited to the OMM under reducing conditions, in contrast to its presumed oxidized state. For quantification of this effect, we chose an in vitro approach (see below).

### Binding constants of GapC1 interacting with VDAC3 under reducing and oxidizing conditions

**Fig. 2:**
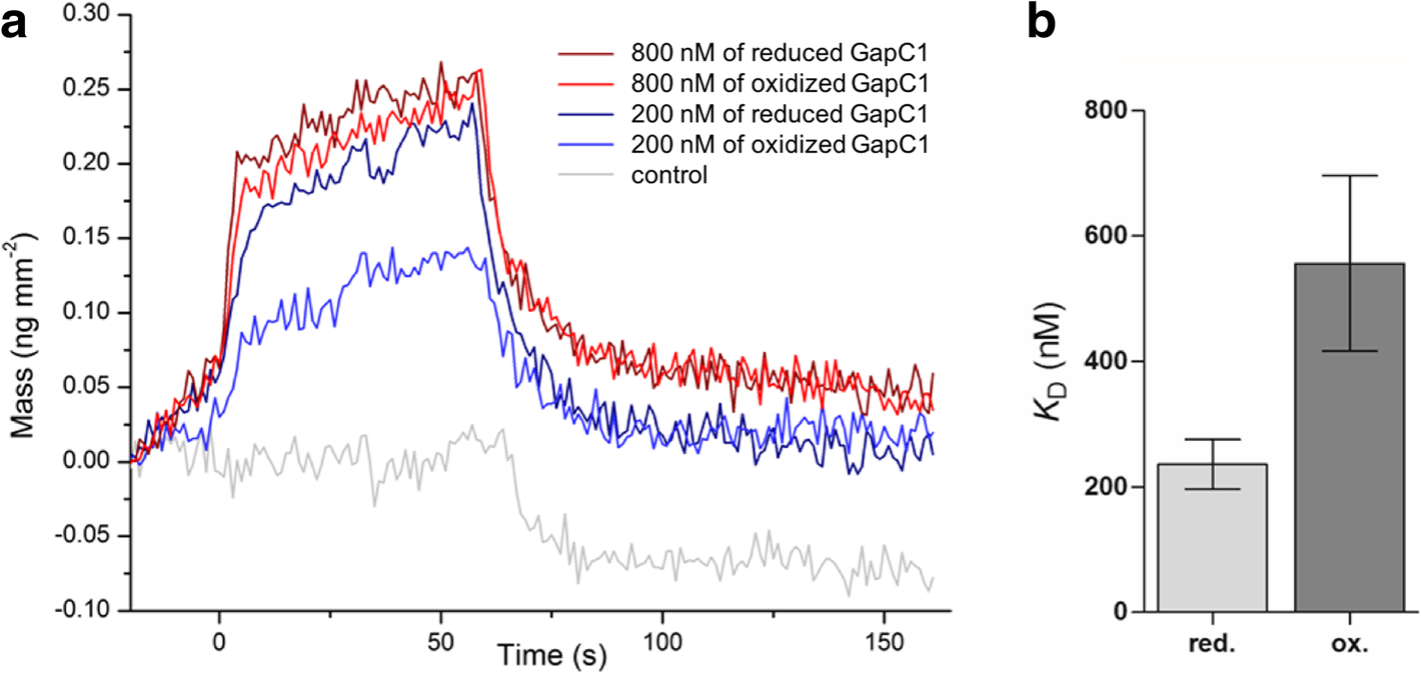
Redox-dependent binding of GapC1 to VDAC3. **a** Association and dissociation behavior of reduced and oxidized recombinant GapC1 added to bilayer-incorporated recombinant VDAC3 at the indicated concentrations is shown as original traces. For reduction, samples were pre-treated with 2 mM DTT, for oxidation with 0.5 mM H_2_O_2_ + 0.5 mM GSH. **b** Equilibrium dissociation constants (*K*_D_) of pre-treated GapC1 binding to VDAC3 were obtained as described in Material & Methods. Error bars represent SD, *p*-value by Students t-test *p* ≤ 0.005

In earlier studies, an association of the GapC protein with the OMM via VDAC3 had been demonstrated in a split-YFP assay with mesophyll protoplasts kept under reducing conditions [9]. In the light of the redox-dependent GapC localization to the OMM (Fig. 1), we decided to determine the binding kinetics of GapC1 to the mitochondrial VDAC3 under reducing and oxidizing conditions by RIf. In this approach, recombinant VDAC had been refolded and incorporated into a lipid bilayer, thus representing the native state on a solid support membrane. Binding and dissociation of GapC1 to reconstituted VDAC3 was recorded in a flow chamber by measuring changes in white-light interference in a mass- and protein-sensitive manner (Fig. 2a, Additional file 2: Figure S1). Different amounts of reduced (pretreatment with 2 mM DTT) and oxidized (pretreatment with 0.5 mM H_2_O_2_ + 0.5 mM GSH) GapC1 protein were injected to determine the redox-dependent effects on the strength of the interaction with the VDAC3 resident in a lipid bilayer (Fig. 2a). Binding properties of GapC1 to a protein-free lipid membrane served as a negative control. Thus, the equilibrium constants (*K*_D_) for the interaction of reduced and oxidized GapC1 with VDAC3 could be determined. The *K*_D_ value of oxidized GapC1 ranged at 556.3 ± 69.9 nM, while a lower *K*_D_ value of 236.0 ± 19.8 nM was obtained for reduced GapC1 (Fig. 2b**,** Additional file 2: Figure S1). This indicates a twofold increase of the affinity for VDAC3 of reduced GapC1 compared to the oxidized form.

### Nuclear localization of GapC isoforms and their cysteine mutants under different redox conditions

**Fig. 3:**
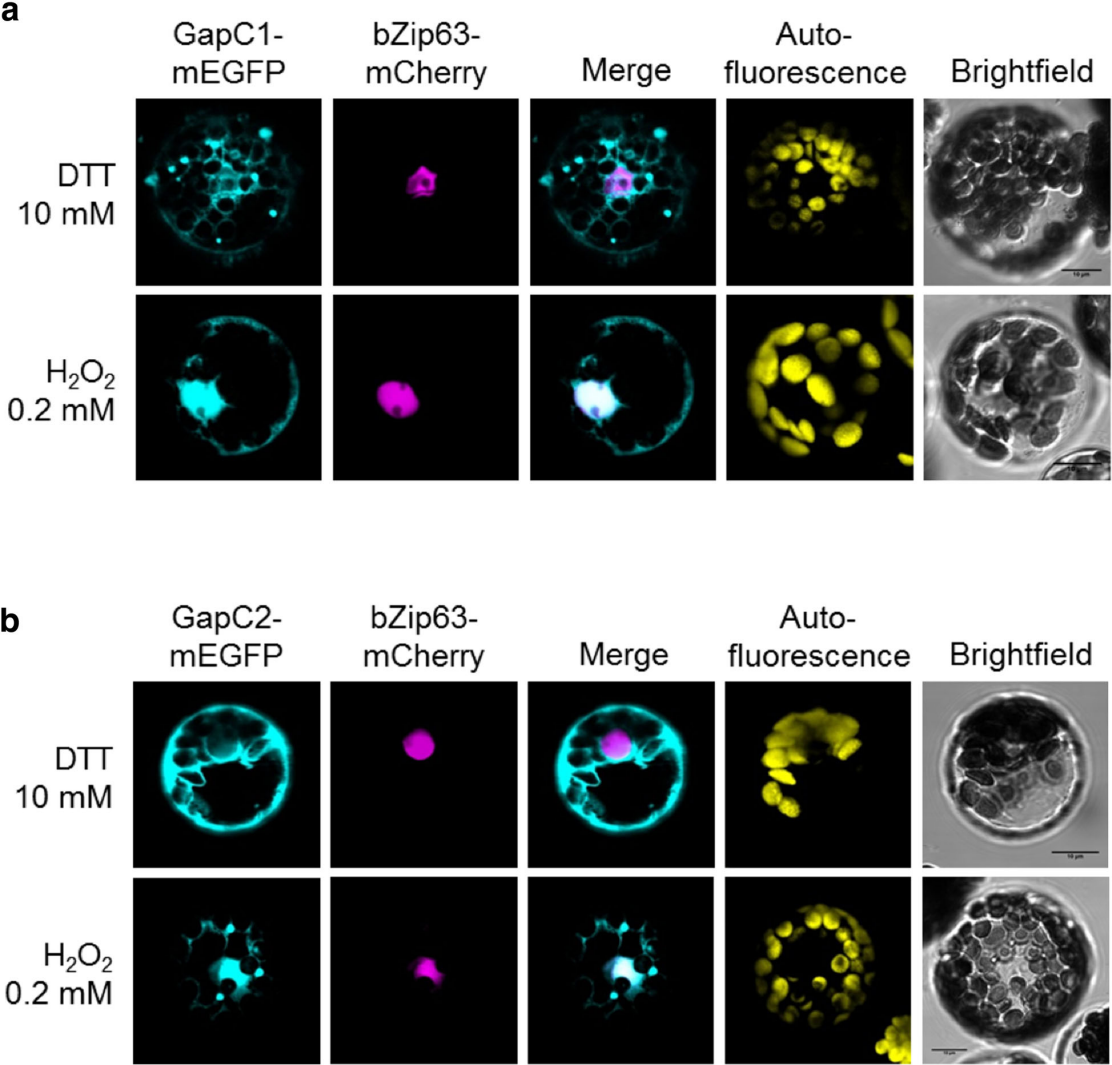
Subcellular localization of GapC under reducing and oxidizing conditions. Isolated mesophyll protoplasts of *A. thaliana* were transiently transformed with (**a**) GapC1-mEGFP or (**b**) GapC2-mEGFP fusion proteins (cyan). The nucleus was labeled with bZip63 fused to mCherry (magenta). Chlorophyll autofluorescence is shown in yellow. Transformed protoplasts were treated with 10 mM DTT or 0.2 mM H_2_O_2_ for 30 min. Nuclear colocalization events are shown in white. The scale bar is set to 10 μm

Besides their interaction with VDACs at the mitochondrial surface, GapC isoforms can also translocate into the nucleus. Therefore, GapC-mEGFP fusion proteins were expressed in Arabidopsis mesophyll protoplasts to follow their subcellular distribution under different redox-conditions. For this purpose, the protoplasts were pretreated with 10 mM DTT or 0.2 mM H_2_O_2_, before analysis by cLSM. Both, GapC1- and GapC2-mEGFP fusion proteins showed fluorescent signals in the cytosol, but also in the nucleus. Nuclear localization was confirmed by the overlap with the fluorescent signals of the nuclear bZip63-mCherry marker (Fig. 3a, b). In protoplasts subjected to 10 mM DTT, the fluorescent signals appeared predominantly in the cytosol, while pretreatment of the protoplasts with 0.2 mM H_2_O_2_ resulted in an increased nuclear localization of GapC1-mEGFP and GapC2-mEGFP fusion proteins (Fig. 3a, b).

**Fig. 4:**
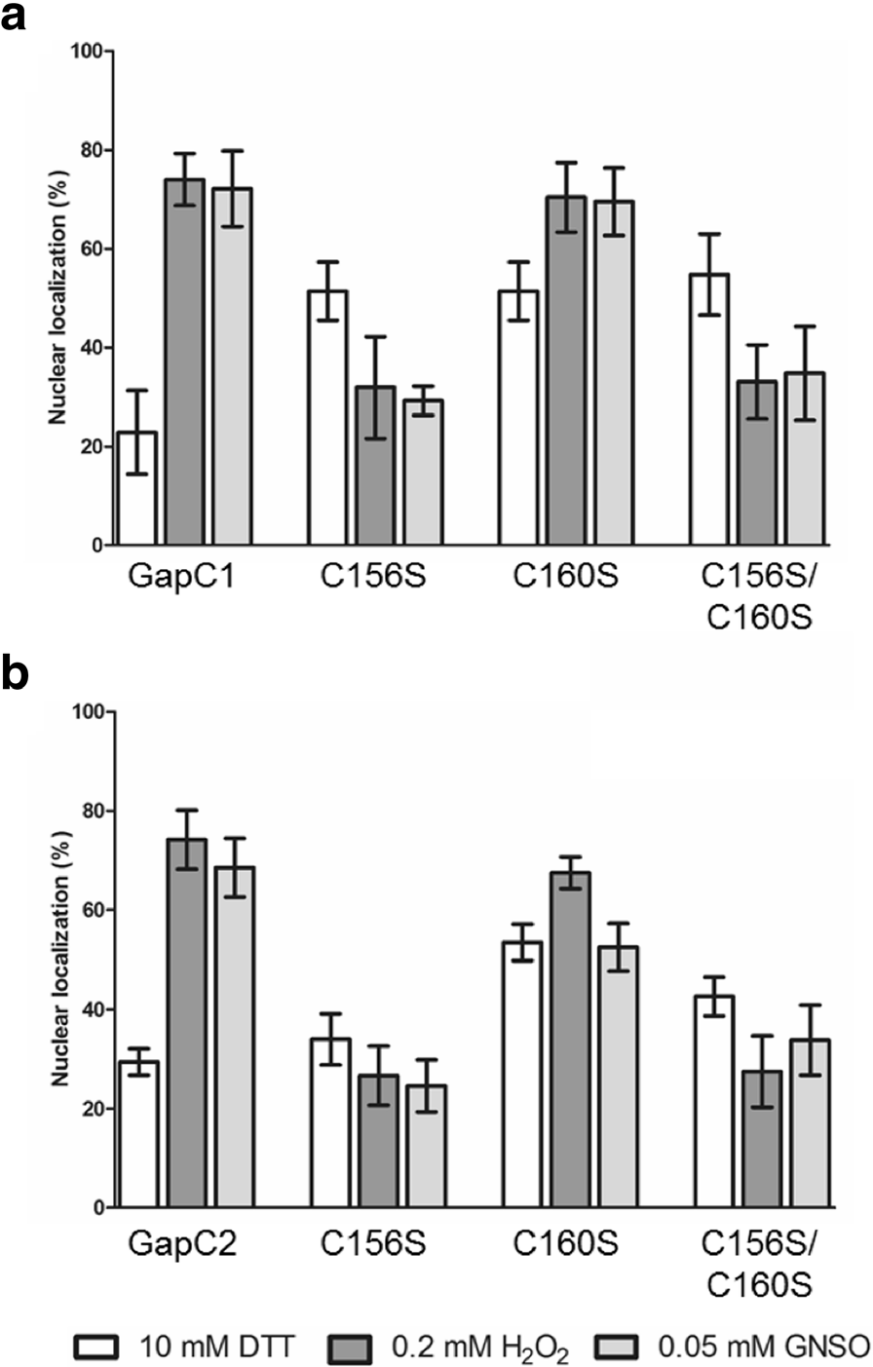
Redox-dependent distribution of GapC between cytosol and nucleus. For quantification of nuclear localization, mesophyll protoplasts were transiently transformed with GapC-mEGFP fusions of the isoforms GapC1 (**a**), and GapC2 (**b**), and their corresponding single (C156S and C160S) and double (C156S/C160S) cysteine mutants. The subcellular localization was determined after the treatment with different redox reagents (10 mM DTT; 0.2 mM H_2_O_2_ or 0.05 mM GSNO). (n ≥ 50 analyzed protoplasts. The sum of cytosolic and nuclear mEGFP-signal corresponds to 100% for each experiment; mean ± SD)

For quantification of the redox-dependent localization of GapC1/2, single and double Cys mutants of GapC1 and GapC2 were also included to determine the influence of presence and redox-state of the key cysteine residues on the nuclear localization of GapC. At least 50 protoplasts were analyzed for subcellular localization of each case. In accordance with the previous observations, wild-type GapC1 and GapC2 showed a significant increase of nuclear localization under oxidizing conditions compared to the reducing conditions (Fig. 4a, b). GSNO was included in this experiment, since ROS can initiate NO formation in vivo leading to the formation of GSNO by nitrosylation of a small portion of the GSH pool. Both thiol oxidants, GSNO and H_2_O_2_, led to similar distributions of the GapC isoforms between cytosol and nucleus. In contrast, protoplasts carrying GapC with either the C156S mutation or the C156S/C160S-double mutation did not respond with an increased translocation of the GapC isoforms to the nucleus upon oxidation. It should be mentioned that under reducing conditions all mutant enzymes tended to be more abundant in the nucleus than their wild-type counterparts. Taken together, these findings suggest that the catalytic cysteine residue C156 is required for sensing the cellular redox state. Its oxidative modification is required to initiate nuclear translocation of the cytosolic GapC isoforms.

### Interaction of GapC with Trx-*h3*

**Fig. 5:**
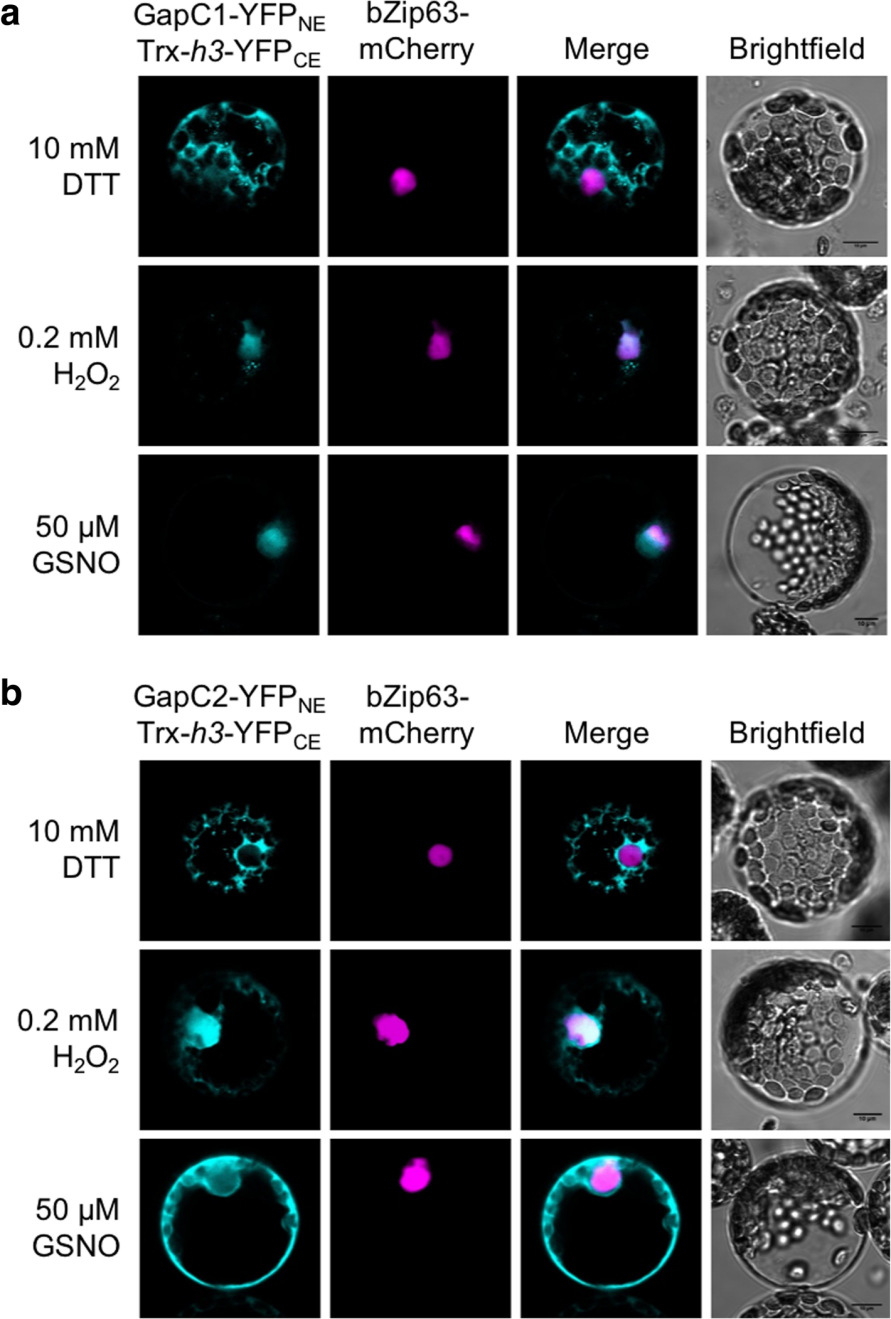
Redox-dependent localization of GapC and Trx-*h3*. Vectors encoding (**a**) GapC1- or (**b**) GapC2-fusion constructs of YFP_NE_, and Trx-*h3*-YFP_CE_ were co-transformed into mesophyll protoplasts. The fluorescent signals from the YFP complementation are shown in cyan. The nuclei are shown in magenta as labeled by bZip63 fused to mCherry. Colocalization events from the complemented YFP and the nuclear-located bZip63-mCherry proteins are shown in white. Transformed protoplasts were treated with 0.2 mM H_2_O_2_ or 10 mM DTT for 30 min. Scale bars were calibrated to 10 μm

GapC has been found as a target of Trx, which can convert the enzyme back into its reduced state [27, 52–54]. Trx, in turn, would be re-reduced by NADPH as mediated by the NTR system [23]. To fulfill its function in vivo, Trx would have to interact with GapC. To test this assumption, split-YFP assays were applied to isolated Arabidopsis mesophyll protoplasts subjected to various redox conditions. The protoplasts were transformed with constructs for fusion proteins of the N-terminal half of YFP (YFP_NE_) fused to either GapC1 or GapC2 and the C-terminal half (YFP_CE_) fused to Trx-*h3*. The bZip63-mCherry nuclear marker was used as a control. Treatment of the transformed mesophyll protoplasts with 20 mM DTT resulted in the interaction of Trx-*h3*-YFP_CE_ with GapC1-YFP_NE_ and GapC2-YFP_NE_, respectively, as indicated by the YFP fluorescence in the cytosol (Fig. 5a, b). Treatment with 0.2 mM H_2_O_2_ or 0.05 mM GSNO to establish an oxidative environment led to enhancement of the nuclear fluorescence signal, generated by the interaction of Trx-*h3* with either GapC1 or GapC2 in the nucleus. Nevertheless, a significant portion of these interactions could still be detected in the cytosol under these conditions (Fig. 5a, b). This result indicates that Trx-*h3* might be able to remove the oxidative modifications at the active-site cysteine C156 of GapC, both in the cytosol and in the nucleus.

**Fig. 6:**
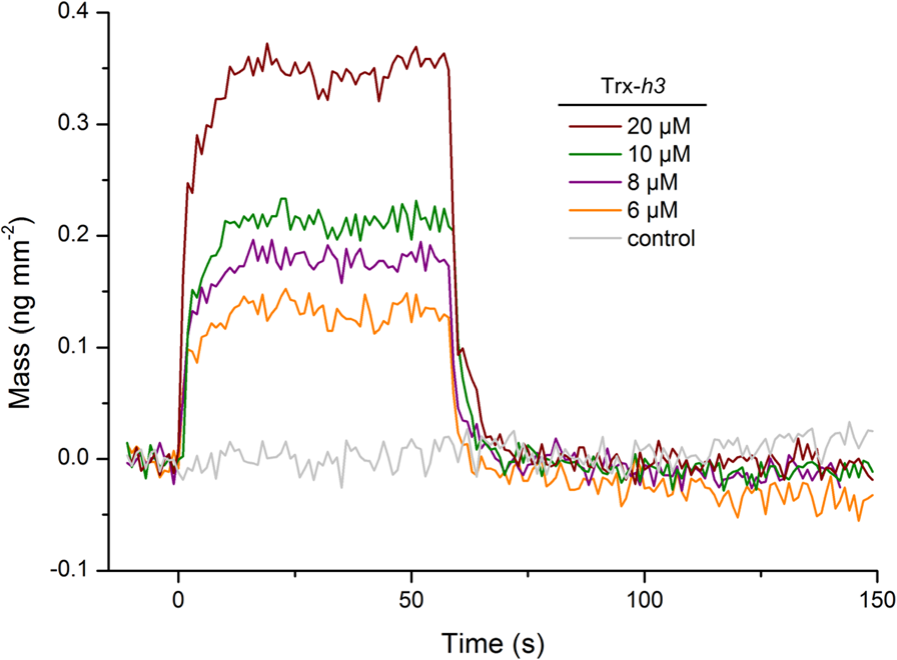
Binding of Trx-*h3* to GapC1. Binding kinetics of Trx-*h3* to immobilized GapC1 were monitored by RIf spectroscopy. The experiment was performed in 20 mM HBS-buffer (100 mM NaCl, 0.01% Triton X-100, pH 7.5) supplemented with 2 mM DTT. The GapC1 protein was immobilized via NHS. Association and dissociation reactions were recorded at different Trx-*h3* concentrations

The interaction of GapC with Trx-*h3* was further analyzed in vitro by RIf. Association and dissociation of Trx-*h3* to the immobilized GapC1 protein was recorded at different concentrations of Trx-*h3* (Fig. 6)*.* Due to the transient interactions, the *K*_D_ had to be determined by the equilibrium binding signals using a Scatchard plot and yielded a value of 40 μM **(**Additional file 3: Figure S2). In contrast, immobilization of the unrelated yellow fluorescent protein mCitrine to the surface did not lead to any significant association of Trx-*h3*.

### Complementation of a yeast *zwf1* deletion strain lacking G6PDH by expression of plant cytosolic GapN

The reduced cofactor NADPH is required to recover cells from oxidative stress. It provides electrons for re-reduction of oxidized glutathione (GSSG) and GSNO, and the reversal of oxidative modifications at GapC by Trx and NTR [22]. Any enzyme or signaling activity of GapC affected by its oxidation, or that of its orthologs from other organisms, can thus be reverted when the cells recover from the stress situation, and the metabolic flux can be switched back to active glycolysis. The oxidative branch of the pentose pathway, with G6PDH as a key enzyme, generates NADPH in all organisms, in human and in yeast cells represented by single isoforms and in plants by six isoforms [55]. In addition, plants and bacteria possess a highly stable GapN [54, 56], which is a prime candidate for additional NADPH production in stressful conditions.

**Fig. 7:**
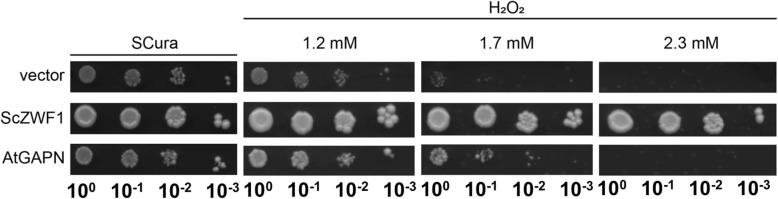
Complementation of a yeast zwf1-deletion strain by plant GapN. Serial drop dilution assays of the yeast strain HOD269-1C carrying the vector without insertion (pJJH2064), the plasmid with the yeast endogenous *ZWF1* gene under the control of the constitutive *PFK2* promoter (pJJH2111), or the plasmid with the GapN-encoding sequence under the control of the *PFK2* promoter (pJJH2118) were performed. Cells were adjusted to an OD_600_ of 0.1 as described in Materials & Methods. Serial dilutions were dropped onto SCura plates containing the indicated concentrations of hydrogen peroxide. Images were taken after incubation for 3 days at 30 °C

To study this putative function of GapN in a system devoid of background activity, we constructed a yeast *zwf1* deletion mutant, which lacks its endogenous G6PDH [57, 58]. As a positive control, we employed a similar construct carrying the native *ZWF1* gene from *S. cerevisiae*. All genes were expressed from a single-copy *CEN/ARS* plasmid under the control of the constitutive *PKF2* promoter, in a derivative of a system previously employed for the heterologous expression of *PFK* genes from patients with Tarui’s disease in yeast [59] introduced into the *zwf1* deletion. Drop-dilution assays of the different transformants on plates containing H_2_O_2_ demonstrated that the *zwf1* deletion carrying an empty vector was highly sensitive (Fig. 7). This sensitivity was not only rescued by the native Zwf1 but also to a considerable extent by the plant enzyme GapN. This result indicates that GapN from plants can generate sufficient NADPH to mediate resistance against oxidative stress conditions even in yeast cells. The weaker suppression of the oxidative stress sensitivity by GapN as compared to the complementation capacity of the native Zwf1 could be attributed to two lines of reasoning: i) the native *ZWF1* gene expressed under the control of the yeast *PFK2* promoter generates an enzyme with a specific G6PDH activity of 303 ± 10 mU/mg protein. On the other hand, specific GapN activity only amounted to 10 ± 2 mU/mg protein, which is near the limitof detection in the assay used and may thus generate much less NADPH than the Zwf1 reaction. ii) Since GapN activity is not present in normal yeast cells, diversion of the flux from the NADH-generating glycolytic reaction of the yeast’s GAPDH isoforms may interfere with the yeast’s metabolism and lead to a reduced capacity of antioxidant defense. Nevertheless, the increased resistance of the transformants with GapN activity as compared to those with the vector control clearly demonstrates its ability to generate sufficient NADPH in yeast to suppress some of the negative effects of H_2_O_2_ exposure.

## Discussion

We here demonstrate evidence that the GapC isoforms of *Arabidopsis thaliana* localize to different cellular compartments depending on their oxidation state. This may be related to moonlighting functions apart from the catalysis of the glycolytic reaction. Thus, active glycolytic metabolons have been suggested to form in the vicinity of the mitochondrial surface. When the redox-state of the cell starts to be shifted into oxidizing conditions upon stress, a portion of the redox-sensitive GapC will be oxidized and can translocate into the nucleus, where it possibly initiates a changed gene expression.

Association of GapC with the outer membrane of plant mitochondria had been shown previously [9, 50, 51]. We observed an enhanced colocalization of GapC1 with mitochondria under reducing conditions when cells are in a metabolically active state compared to oxidizing conditions. This indicates the formation of metabolons by which glycolysis is directly taking place in proximity to mitochondria. Such metabolons are built of glycolytic enzymes, channeling the intermediates efficiently through the pathway and enabling import of the products into mitochondria for further oxidation in the respiratory chain. VDAC might serve as a docking platform for such metabolons. The present in vitro interaction study revealed that reduced GapC exhibits a higher affinity for VDAC compared to the oxidized GapC. In fact, this supports the hypothesis that reduced GapC forms an active glycolytic metabolon attached to the OMM via VDAC3.

In colocalization experiments presented here, GapC was found to accumulate in the nucleus upon oxidative stress. Furthermore, GapC had been detected in nuclei of cadmium-treated roots [10], and in calcium- and NO-stressed tobacco BY-2 cells [11]. There is now a growing number of publications describing the “moonlighting” functions of GAPDH in the nucleus [2, 3, 5, 16]. As a well-studied example in the mammalian system, an association of *S*-nitrosylated GAPDH and SIAH1, an E3-ubiquitin ligase, leads to nuclear translocation and induction of cell death [60]. In fact, a similar protein, namely SINAL7, was identified as a binding partner of GapC in plants [61]. In rice, GapC was found to be responsible for transcriptional activation of glycolytic genes under oxidative stress [62].

In various Trx-interaction studies, GapC had been found as a target protein [27, 52]. Due to the transient nature of the nuclear localization, the interaction with a redox-mediating compound such as Trx must be assumed for the reversal of the cysteine modifications. Trx type-*h* was found in the nucleus of oxidatively stressed Arabidopsis cells [63, 64]. It had been suggested that denitrosylation of modified proteins in animal cells is catalyzed by the thioredoxin system [65]. In Chlamydomonas, cytosolic Trx-*h* isoforms even seem to possess some specificity of their nuclear functions in response to different types of DNA damage [64]. If GapC fulfills a moonlighting function by regulation of nuclear gene expression, interaction with Trx-*h3* could be interpreted in view of the reversal of the oxidative modification and concomitant export from the nucleus, thus aborting its access to the transcription machinery after a stress period.

**Fig. 8:**
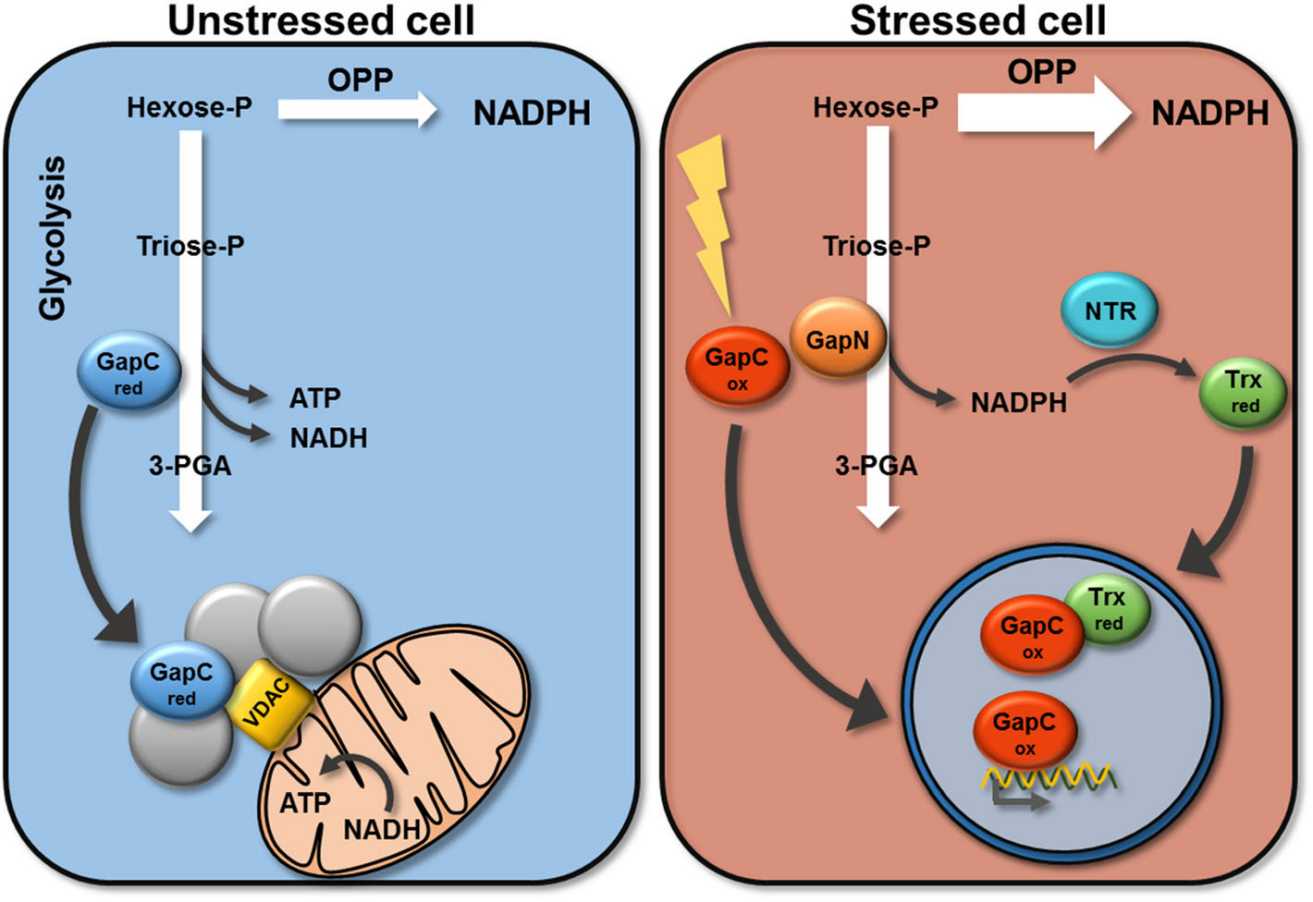
Pathways of carbohydrate oxidation. In unstressed cells (left panel), glycolysis and respiration are reinforced by the formation of a metabolon at the mitochondria attached to VDAC. In a stressed cell (right panel), GapC serves as a redox-sensor and mediates signal transfer to the nucleus, where genes required for poising and ROS defense are induced. NADPH is provided by GapN, specific for plants. In both cases, NADPH can also be provided by the OPP

As soon as any disturbance of the metabolic flux becomes evident, H_2_O_2_ or NO is formed leading to modifications of the most sensitive thiols of GAPDH [13, 66], triose-phosphate isomerase [67] or pyruvate kinase M2 [68]. These examples demonstrate that the central glycolytic pathway is flexibly adjusted by linking the cellular redox-state and the substrate pool sizes at multiple steps. Due to the concomitant inactivation upon oxidative stress, glucose oxidation is redirected towards the OPP for NADPH generation by G6PDH [69]. Alternatively, the oxidation of GAP via GapN is an additional source for NADPH in plants, especially under increasing oxidant levels [54] (Fig. 8).

Due to its redox-sensitive catalytic cysteine, GapC provides a connection of redox-state and energy metabolism [13, 16]. Therefore, GapC might serve as a central hub for energy supply and signal transfer to execute the various cellular fates [70, 71]. Redox-dependent processes are now accepted as major players in a multitude of cellular processes, and as important signal transmitters to induce readjustment of homeostasis upon oxidative stress [72]. Various sources, duration, and levels of stress can be transmitted by a specific post-translational modification code that is highly dynamic. Stress stimuli could lead to a differential modification pattern on a single protein. The idea of a modification code combining modifications also of other residues with the various redox-modifications of cysteine is tempting. Obviously, such a code can only be investigated and deciphered in vivo. Yet, only a minor proportion of each protein in question is expected to be modified for triggering the specific response. To solve this problem, high-resolution analysis of all single GAPDH molecules would be required to identify different modification patterns in subfractions of the cellular GAPDH pool.

An equally challenging task is the exact determination of the stimuli that provoke each modification, resulting in a complex interplay of different factors in vivo, and different output in each case. These modifications are additionally dependent on metabolites, microenvironmental conditions and/or macromolecular crowding effects [73, 74]. To prove any effect in such a complex scenario in vivo and to mimick a realistic microenvironment in vitro*,* will be a difficult task for the future. The investigation of the switching ability between GAPDH activity in the central metabolism and its role as a redox-sensor by classical genetic methods carries the intrinsic problem that these functions are both dependent upon the active-site cysteine of the protein. GAPDH-knockout mutants show many pleiotropic effects which are not distinguishable in relation to their origin due to the dual role of the active-site cysteine. Any effects could be either caused by the lack of the moonlighting functions of GAPDH or an impeded central metabolism when glycolysis is not functional.

## Conclusions

The glycolytic enzyme GapC is a versatile hub to sense and counteract metabolic and redox imbalances. Both pieces of information are integrated into signaling pathways for a proper response. Many different input signals evoke multiple cellular responses, ranging from improved growth to induction of programmed cell death (PCD). In particular in plants, integration of signals from environmental impact and developmental state in the various photoautotrophic and heterotrophic tissues requires effective regulatory systems that respond accordingly to maintain homeostasis [75]. The occurrence of *S*-glutathionylation, *S*-sulfhydration, and/or *S*-nitrosylation at the catalytic cysteine and/or the neighboring cysteine of GAPDH could serve as a redox code [76]. The degree and kind of stress might even be reflected in a specific pattern of the various possible modifications of sensitive cysteines, thus transferring the actual information in a finely graduated way to obtain the proper output. Based on such a system, a wide spectrum of environmental challenges and metabolic imbalances can be responded to at the various levels of regulation to regain and maintain homeostasis. In the worst case, execution of PCD to prevent further ROS formation in damaged cells, when metabolism gets out of control, might then be also an appropriate response.

A transient inactivation of GAPDH suggests that the glycolytic pathway will be down-regulated during oxidative or nitrosative stress. For the time of imbalance, this leads to a redirection of the metabolic flux away from glycolysis and ATP production into the OPP pathway for rapid generation of NADPH required for GSSG reduction poising, and repair [69]. Additionally, apart from the oxidation of G6P through the activity of G6PDH, the required NADPH can be provided by oxidation of triose-P through the cytosolic GapN. Ectopic expression of GapN enables the yeast strain lacking G6PDH to persist oxidative stress conditions by its capability to provide NADPH (Fig. 8).

On the other hand, oxidative modifications affect the binding properties of GapC and its subcellular localization. Due to the various post-translational modifications, transient formation of subcompartments or metabolons, changing cellular localizations and moonlighting functions can be initiated [77]. Upon regain of homeostasis, signals need to be converted back to normal, and regular energy metabolism should recover. This requires the reversal of GapC modifications and ending of its moonlighting functions. Here, the NADPH-generating systems for regeneration of reduced thioredoxins and reversal of cysteine modifications are also required.

In the present study, we could demonstrate the redox-dependence of the cellular localization of GapC, resulting in increased nuclear localization of both isoforms under oxidizing conditions, and the interaction with Trx-*h3* in the nucleus. Binding of GapC isoforms to the mitochondrial VDAC, on the other hand, was more pronounced under reducing conditions as would be the case upon recovery. The efficiency of the glycolytic flux is then optimized by the formation of a metabolon in close vicinity of the mitochondria.

## Additional files

Additional file 1: **Table S1.** Primer used for the amplification of the *ZWF1* gene encoding the glucose-6-phosphate dehydrogenase. Primer sequences used to generate the different yeast strains for the yeast complementation assay. (DOCX 14 kb)

Additional file 2: **Figure S1.** Redox-dependent binding of GapC1 to VDAC3. Binding kinetics of the GapC1-VDAC3 interaction were monitored by RIf spectroscopy. (**A**) After the injection of VDAC3-containing proteoliposomes (gray), the formation of a polymer-supported membrane and the binding of GapC1 to VDAC3 (light blue) were recorded by label-free RIf detection. (**B**) Representative association and dissociation behavior of reduced and oxidized GapC1 and VDAC3 with the respective residuals. (PPTX 169 kb)

Additional file 3: **Figure S2.** Determination of the *K*_*D*_ for the interaction of GapC1 with Trx-*h3*. The values of the equilibrium binding signals (Γ_eq_) for each experiment were plotted against the ratio of the binding signal to the concentration of Trx-*h3* (Γ_eq_/c). The *K*_*D*_ value of the corresponding interaction of Trx-*h3* with GapC1 was extracted from the slope of the corresponding. (PPTX 50 kb)
