# Supplementary material for: Cytosolic GAPDH as a redox-dependent regulator of energy metabolism
Source: BMC Plant Biol. 2018 Sep 6;18:184. doi: 10.1186/s12870-018-1390-6 (PMC6127989; doi:10.1186/s12870-018-1390-6)
Supplement: Supplementary file 1 — Table S1. Primer used for the amplification of the ZWF1 gene encoding the glucose-6-phosphate dehydrogenase. Primer sequences used to generate the different yeast strains for the yeast complementation assay. (DOCX 14 kb) [file 12870_2018_1390_MOESM1_ESM.docx]

**Table S1: Primer used for the amplification of the ZWF1 gene encoding the glucose-6-phosphate dehydrogenase.** The primer sequences used to generate the different yeast strains for the yeast complementation assay are listed.

| Primer identifier | Sequence 5‘ – 3‘ |
| --- | --- |
| 16.232 | GCGTGAGCTCCTGGTAAGTAAGGTGTAGTTTTG |
| 16.233 | GTGAGTCGACGATAAGTACAAGTCCAATCGGACTG |
| 16.234 | GGCGGGATCCATGAGTGAAGGCCCCGTCAAATTCG |
| 16.235 | ATGAGTGAAGGCCCCGTCAAATTCGAAAAAAATACCGTCATATCTTCGTACGCTGCAGGTCGAC |
| 16.236 | CTAATTATCCTTCGTATCTTCTGGCTTAGTCACGGGCCAAGCATAGGCCACTAGTGGATCTG |
