# Supplementary figures and images for: Cytosolic GAPDH as a redox-dependent regulator of energy metabolism
Source: BMC Plant Biol. 2018 Sep 6;18:184. doi: 10.1186/s12870-018-1390-6 (PMC6127989; doi:10.1186/s12870-018-1390-6)

## Slide 1
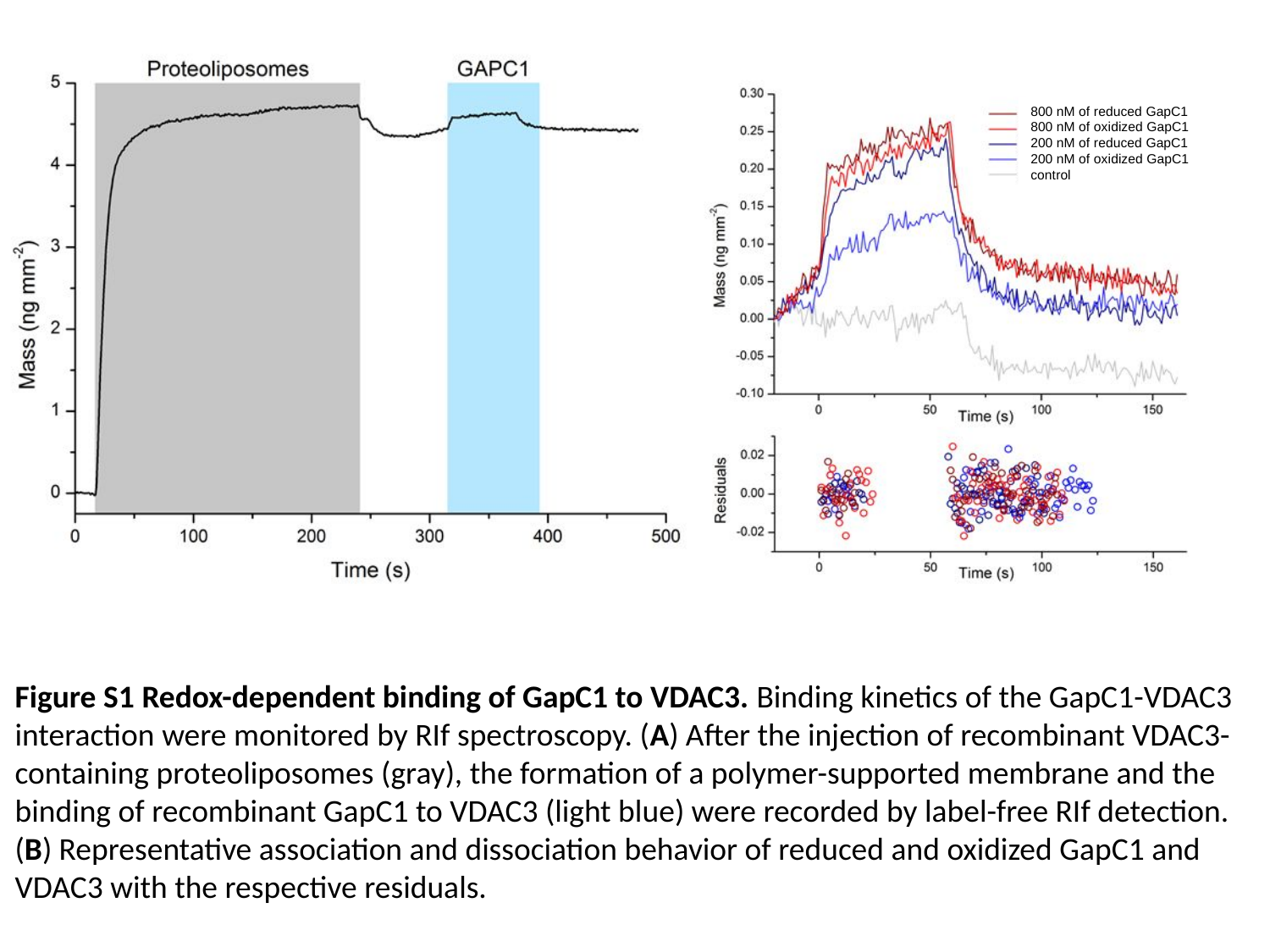

Supplement: Supplementary file 2 — Figure S1. Redox-dependent binding of GapC1 to VDAC3. Binding kinetics of the GapC1-VDAC3 interaction were monitored by RIf spectroscopy. (A) After the injection of VDAC3-containing proteoliposomes (gray), the formation of a polymer-supported membrane and the binding of GapC1 to VDAC3 (light blue) were recorded by label-free RIf detection. (B) Representative association and dissociation behavior of reduced and oxidized GapC1 and VDAC3 with the respective residuals. (PPTX 169 kb) [file 12870_2018_1390_MOESM2_ESM.pptx]

## Slide 1
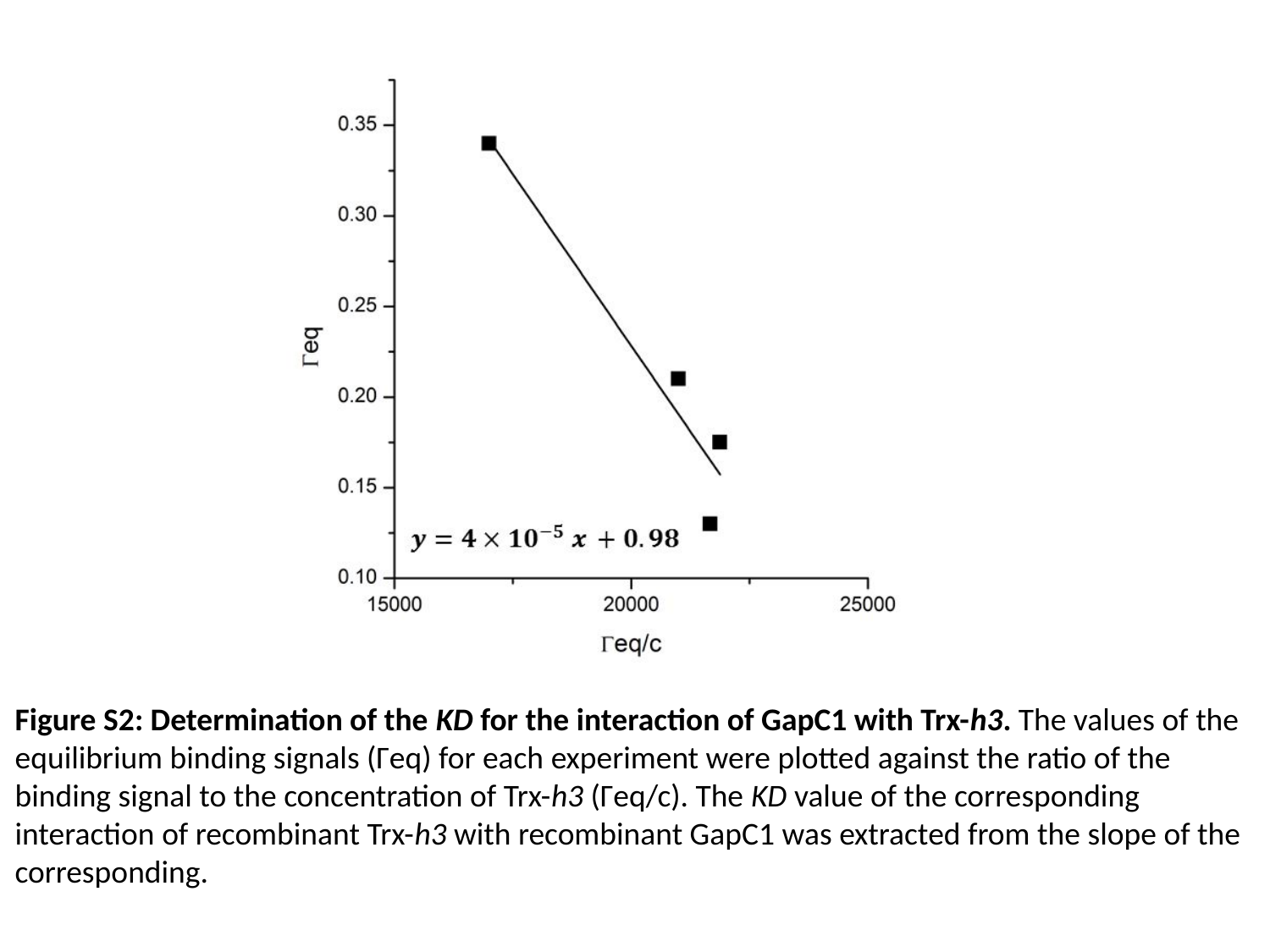

Supplement: Supplementary file 3 — Figure S2. Determination of the KD for the interaction of GapC1 with Trx-h3. The values of the equilibrium binding signals (Γeq) for each experiment were plotted against the ratio of the binding signal to the concentration of Trx-h3 (Γeq/c). The KD value of the corresponding interaction of Trx-h3 with GapC1 was extracted from the slope of the corresponding. (PPTX 50 kb) [file 12870_2018_1390_MOESM3_ESM.pptx]
